# Validation of a mobile game-based assessment of cognitive control among children and adolescents

**DOI:** 10.1371/journal.pone.0230498

**Published:** 2020-03-20

**Authors:** Hyunjoo Song, Do-Joon Yi, Hae-Jeong Park

**Affiliations:** 1 Department of psychotherapy, the Graduate School of Professional Therapeutic Technology, Seoul Women's University, Seoul, Republic of Korea; 2 Department of Psychology, Yonsei University, Seoul, Republic of Korea; 3 Graduate Program in Cognitive Science, Yonsei University, Seoul, Republic of Korea; 4 Department of Nuclear Medicine, Department of Psychiatry, Yonsei University College of Medicine, Seoul, Republic of Korea; 5 BK21 PLUS Project for Medical Science, Yonsei University College of Medicine, Seoul, Republic of Korea; Universtiyt of Oviedo (Spain), SPAIN

## Abstract

Cognitive control is the most fundamental psychological function that underlies the execution of many other psychological functions. A mobile game application could be a useful strategy to evaluate cognitive control in the groups of children and adolescents. Although a serious game that is based on gamification would be an optimal platform for the administration of behavioral and clinical assessments of children and adolescents, most studies on gamification have been conducted among adults and older adults than among children and adolescents. This study aimed to assess cognitive control using a mobile game that used gamification and compared the results to those from traditional neuropsychological tests for children and adolescents. In order to address this objective, this study used a serious game, namely, “CoCon,” which was developed to assess cognitive control in children and adolescents. This study included 100 participants from a community sample (mean age = 11.75 years, ranged from 9 to 16 years, *SD* = 1.40 years; Male = 59(59%), Female = 41(41%)). The analyses interrogated the relationships among various game behaviors scores of CoCon, the standardized neuropsychological tests (K-WISC-IV, CTT, and Stroop), and self-reporting executive function difficulty questionnaire. As results, a mobile game application-based assessment proved to be a reliable and valid measure of the cognitive control in children and adolescents. The index scores from the CoCon were significantly related to various cognitive control functions and differentiated between the high and low cognitive control groups. Specifically, even though the participants completed the mobile game ‘CoCon’ in their natural habitats, the CoCon scores were comparable to the measures from standard neuropsychological tests. In conclusion, the present findings suggest that mobile games that use advanced technology and sophisticated psychological strategies can serve as a new and expanded platform for the administration of psychological assessments.

## Introduction

Advancements in mobile devices across the past decade have tremendously transformed lifestyle in modern society. Adolescents who are between the ages of 18 and 24 check their smartphones as often as 86 times a day [1]. Mobile applications have emerged as the core components that have led to these tremendous changes, and various health-related fields are rapidly embracing the use of these technologies in their research investigations. Several investigators in these fields have developed new intervention strategies that use mobile phone applications and have found them to be effective [2–6]

Cognitive control is the most fundamental psychological function that underlies the execution of many other psychological functions. Gratton, Cooper, Fabiani, Carter, and Karayanidis [7] described that cognitive control is a core concept in modern cognitive neuroscience. Besides, it described that cognitive control is the set of mechanisms that are arranged in tasks demanding flexibility at one or multiple levels. Miller [8] has described cognitive control as the mechanisms that orchestrate behavior in accordance with one’s behavioral intentions in complex environments, and it is essential for engagement in intelligent behavior. In particular, cognitive control is deeply connected to various behavior problems like ADHD, conduct disorder, and self-injurious or other impulsive behaviors among children and adolescents. From a perspective of Neurobiology, the prefrontal cortex, which is directly or indirectly connected to the sensory areas and motor areas and even subcortical structures [8–10], is the core brain area that is responsible for cognitive control. Further, the neural mechanism that underlies cognitive control is characterized by extensive interconnectivity.

As a consequence of this characteristic feature of cognitive control, it is difficult to theoretically conceptualize and empirically examine what cognitive control is. The debate on whether cognitive control can be regarded as comprising discrete control components or as an emergent feature from other basic psychological functions is still unsolved. For example, Mackie, Van Dam and Fan [11] proposed the attentional theory model of cognitive control and showed attentional function’s predictive value to some extent. However, Mackie’s study [11] didn’t present strong findings to support the attentional function theory of cognitive control. Gratton, et al. [1] contended that since cognitive control is a complex and multifaceted concept, it is difficult to conceptualize it as a unitary integration of its various dimensions. Consequently, although some controversies exist, it was concluded that a current assumption would lead to conceptualize cognitive control as a comprehensive function encompassing from the basic attention to the executive function. Therefore, this study decided to adopt a relatively comprehensive concept of how to define cognitive control.

In particular, it might be possible that the advancement of technology provides a more reliable and valid method to evaluate cognitive control in a broad context. At present, numerous applications are being developed, and clinicians who are experts in the field of human behavior have many opportunities to use these latest and advanced technologies. Accordingly, clinicians can use mobile devices as a platform for the administration of psychological interventions because it affords them more opportunities to use new methods that were impossible to use in the past. In particular, the administration of psychological assessments through mobile devices may overcome the limitations of conventional assessment methods (e.g., poor ecological validity). Chaytor, Schmitter-Edgecombe and Burr [12] insisted that various environmental variables appear to affect the ability of neuropsychological tests to predict real-world behavior and should be studied in future research on ecological validity. In addition, they suggested that research needs to go beyond the tests themselves and attempt to empirically investigate the complex relationships to improve the ecological validity of an executive functioning evaluation. In this context, the mobile application-based assessment could be a good method of attempts to improve the ecological validity. For example, mobile application-based assessments can be administered in natural settings and in the absence of a human expert’s observation.

One of the advantages of mobile application-based assessment is that it allows researchers to collect data from human beings in their natural ecological settings that are not constrained by physical boundaries; therefore, data may enhance the ecological validity of findings. Holmlund et al. [9] have contended that the administration of behavioral assessment through mobile devices is a new direction for behavior assessments in settings that are situated outside the controlled environment of a laboratory. They have also suggested that new mobile application-based psychological assessments have scientific and clinical value because they can be used to explore the temporal dynamics that underlie human behavior and foster a holistic understanding of individual differences in multiple behaviors. Muriel and Crawford [13] have proposed that (video) games offer choices to those who play them, and this feature allows clinicians and researchers to examine the nature and forms of agency within a wider culture.

In particular, the application of computational modeling method using large data that are collected through applications can provide new perspectives that can be used to understand and classify human behaviors. Bunian, Canossa, Colvin, and El-Nasr [14] modeled individual differences and generated behavioral features based on video gameplay data using Hidden Markov model. Although Bunian et al. [14]’s final results may not be considered to be fascinating, their methodological approach merits consideration and further investigation.

A gamification which is typically used in mobile applications can be used to successfully administer psychological assessments through the mobile devices. Gamification and serious games are different concepts. Specifically, gamification is a means to alter behavior, whereas a serious game is a functional game that utilizes gamification. Nevertheless, these two terms have been frequently considered to be synonymous. Gentry et al. [15] have suggested that both concepts share the same aim, namely, to promote user motivation and increase the frequency and duration of interactions. Furthermore, Mandryk and Birk [16] have noted that digital game play yields various types of data that can be used to produce new digital health-related biomarkers. They have proposed that there are five categories of biomarkers: behavior, cognitive performance, motor performance, social behavior, and affect. According to them, these biomarkers are associated with the playing of the off-shelf digital game in ones’ natural habitat, and they could be regarded as indicators of mental health. They have also cautioned that various factors like pharmacological interventions and ecological variables may compromise the predictive power of game-based digital biomarkers.

The word “game” is more familiar to children and adolescents than adults and older adults. Therefore, gamification may be an effective strategy using which psychological assessments can be administered to children and adolescents. Although a serious game that is based on gamification would be a more optimal platform for the administration of behavioral and clinical assessments of children and adolescents, more number of studies on gamification have been conducted among adults and older adults [17] [18] than among children and adolescents. Several studies have demonstrated the effectiveness of gamification in the fields of education and psychological intervention [19] [20]. Areces, Rodríguez, García, Cueli, and González-Castro [21] reported that a continuous performance test based on virtual reality offered a differential diagnosis of ADHD presentations. Moreover, Sood, Toornstra, Sereno, Boland, Filaretti, and Sood [22] reported that their digital application can be used to detect, monitor, and manage dyslexia among 4–8-year-old children. Children tend to experience boredom which they are subjected to long psychological assessments. This in turn interferes with the validity and reliability of the test results [23]. Additionally, children’s behaviors may vary substantially across different circumstances. For example, even children with ADHD can be cooperative and attentive in some testing situations.

According to the conclusions of Cheng, Davenport, Johnson, Vella, and Hickie [24]’s systematic review of articles on gamification, the most commonly targeted mental health problems are anxiety disorder and a poor wellbeing, whereas the least commonly targeted mental health problems are conduct disorder, bipolar disorder, self-injurious behaviors, schizophrenia, and ADHD. They have also observed that gamification may serve as a new means by which human behavior can be changed (i.e., when compared to the traditional approach). However, in the present study, gamification was used in a manner that is consistent with existing frameworks on behavior change (e.g., enhancing motivation, positive reinforcement). A more fundamental problem is that an insufficient number of studies have examined the reliability and validity of mobile application-based assessments. McMillan, Hickey, Patel, and Michell [25] analyzed a total of 223 applications that were being used within the national health system of the United Kingdom, and systematically reviewed a final pool of 49 applications. They found that, although a number of mobile applications were developed and were being developed, studies that had examined the efficacy and feasibility of mobile applications were limited.

Collectively, an application-based assessment that utilizes gamification is suitable for use with children and adolescents. However, only a small proportion of academic reports have documented the effectiveness of mobile application-based assessments that use gamification among these demographic groups [26][27]. In particular, gamification is a very useful method to evaluate and intervene in behavioral problems among children and adolescents, but the efficacy study of mobile application-based assessments that use gamification to target conduct disorder, self-injurious behaviors, and ADHD was not reported, even though conduct disorder, self-injurious behaviors and ADHD are mostly related to dysfunctional cognitive control and are more common among children and adolescents [24].

In this regard, the present study aimed to assess cognitive control using a mobile game that used gamification and compare the results to those that are yielded by traditional neuropsychological tests among children and adolescents. In order to address this objective, we used a serious game, namely, “CoCon,” which was developed to screen cognitive control among children and adolescents. CoCon adopted a narrative game format that imitates commercial games like “room escape games” and “catch the thief.” It consisted of a total of six games that assessed sustained attention, working memory, inhibition and response selection, and categorization using popular and general cognitive experimental paradigms such as visual search, Stroop test, flanker task, and categorization. CoCon was developed as a screening tool to detect potential high-risk users who have difficulties in exercising cognitive control (e.g., impulsivity, inattention, inhibition failure, difficulties in organizing and planning). CoCon was designed to be used in all ecological settings. Therefore, various environmental factors like frequent interruptions and a noisy background can influence performance. However, since unique algorithms, which were developed based on the adaptive staircase procedure, were used in CoCon, they may be effective in reducing environmental disturbances and collecting data that accurately represents the cognitive control of the user. In addition, CoCon was designed to assess ecological behaviors like incomplete task exits, number of logins, and frequency with which game achievements were checked.

In summary. it was hypothesized that the scores that are yielded by CoCon and standard neuropsychological tests of cognitive control are going to show significant relationships. In addition, it was expected that groups of individuals with high and low levels of cognitive control will significantly differ in scores that are yielded by the CoCon based on gaming behaviors. Finally, we hypothesized that some gaming behaviors, namely, ecological behaviors, will be significantly related to performance on standard neuropsychological tests of cognitive control. Ultimately, the study was intended to offer empirical evidence for the use of mobile game applications in screening children and adolescents who might have the vulnerability to cognitive control in a general situation like a home or a school. For this goal, we intended to show a possibility that CoCon could work as a feasible tool to screen cognitive control vulnerability in the groups of children and adolescents at an earlier stage.

## Methods

### Participants

The participants of this study were 100 children (male participants = 59%, female participants = 41%) who were recruited from Seoul and Kyeoung-gi area. These participants were recruited through the internet, and from churches or child and adolescent community centers. They participated in this study on a voluntary basis after they were provided with adequate information about this study’s objectives and procedures through written documents and explanations, which were rendered by research assistants. Each participant was paid 10,000 KRW (corresponding to about $9–10) as compensation for their participation. This study was approved by the institutional review board of Seoul Women’s University (SWU IRB-2017A-66). Written informed consent was obtained from participants as well as their parents.

**Fig 1:**
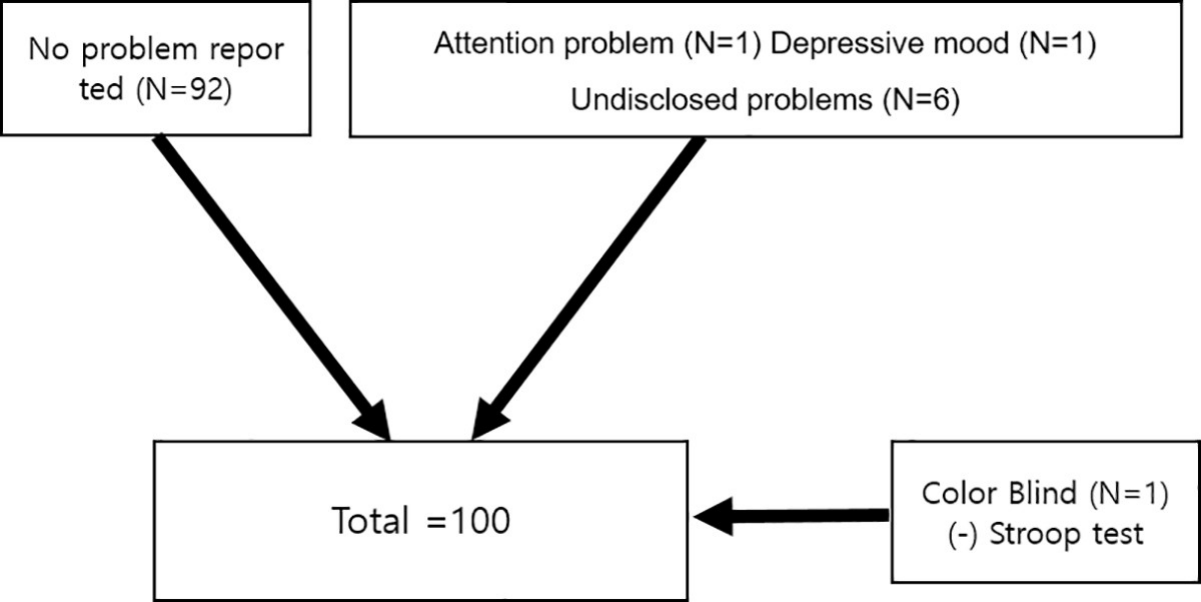
Flow chart of participants.

The ages of the participants ranged from 9 to 16 years (*M*_age_ = 11.75, *SD* = 1.40). Their school grades ranged from the 4^th^ to 9^th^ grade. Out of a total of 100 participants, 8 participants reported that they had received psychological interventions for attention problems (*n* = 1), depressive mood (*n* = 1), and other undisclosed problems (*n* = 6). One participant could not finish the Stroop test because he was color blind; due to which, the missing values were replaced with serial means. Thus, the final sample consisted of 100 participants (Fig 1).

### Instruments

#### Mobile game application ‘CoCon’

**Fig 2:**
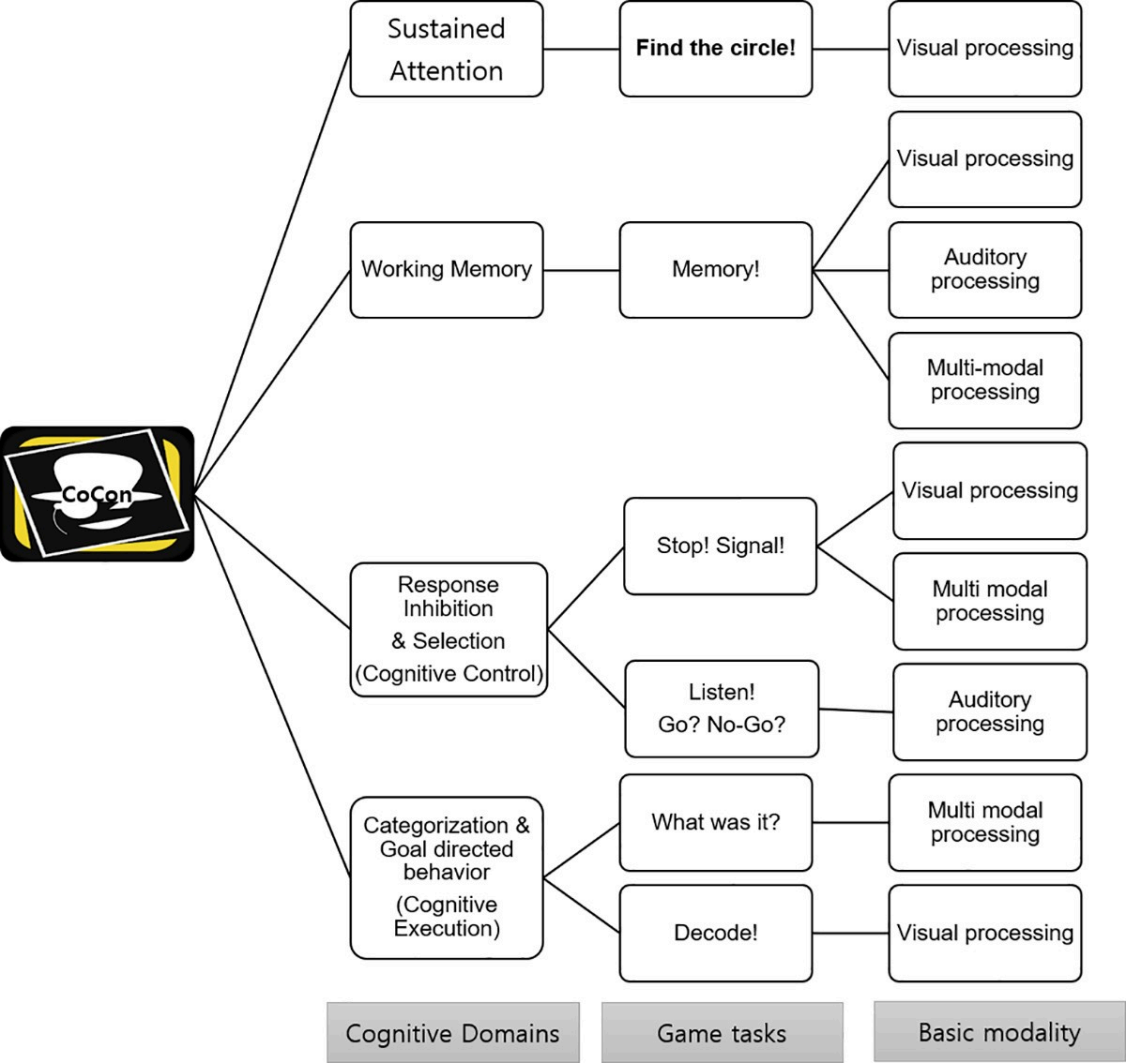
A schematic representation of the contents of CoCon.

**Fig 3:**
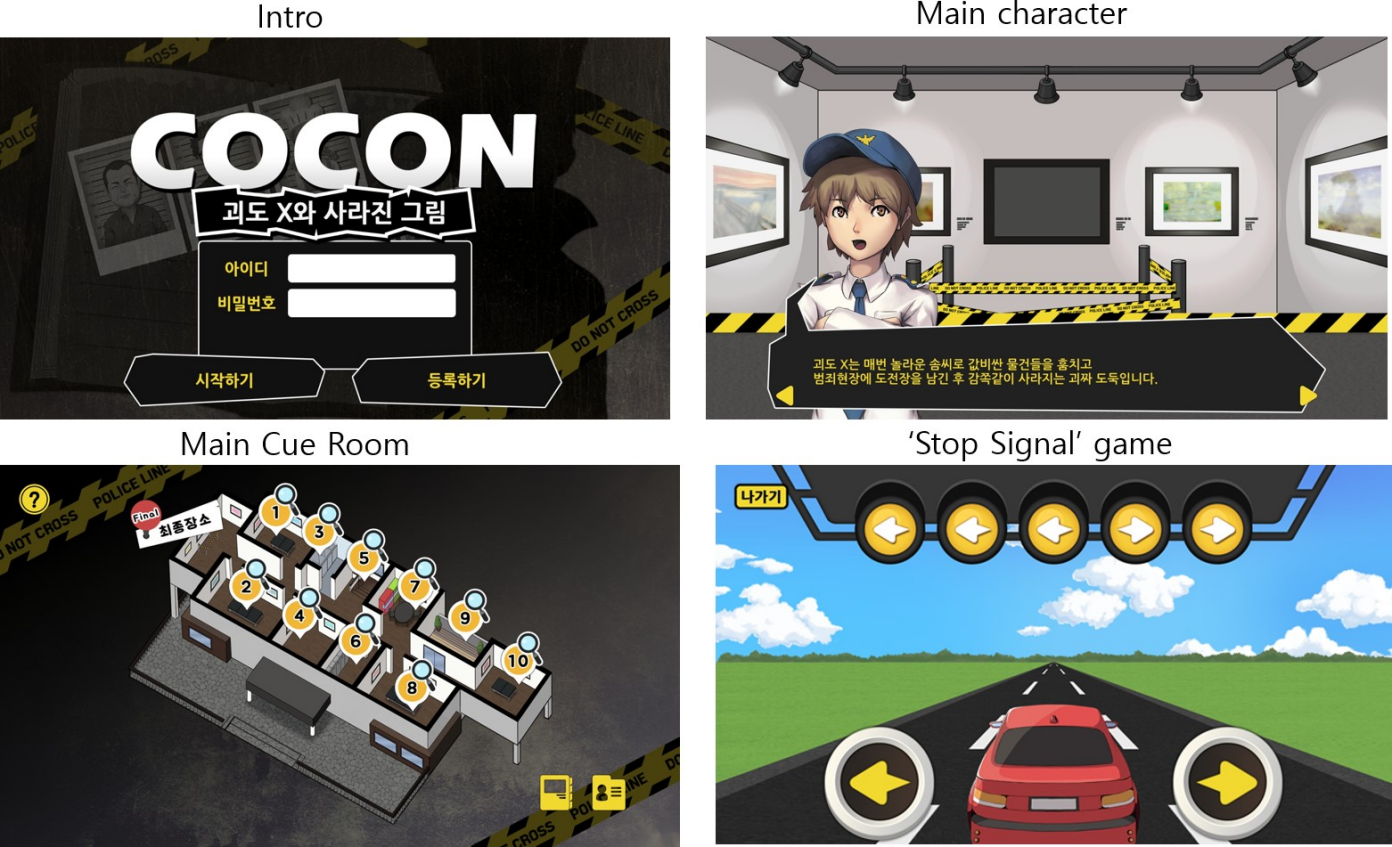
Game images of CoCon.

CoCon tasks were designed to be based on popular general cognitive experimental paradigms such as visual search [28], Stroop [29], flanker [30], and categorization tasks [31]. CoCon entails a narrative game format that imitates entertainment games. CoCon includes a story in which a detective tries to investigate a thief who is hiding in a gallery. The cognitive control abilities of the users are simultaneously assessed while they play this game. A total of six games are included in CoCon. They assess sustained attention (‘Find the circle’), working memory (‘Memory’), inhibitory ability and response selection (‘Stop! Signal’, ‘Listen, Go? NoGo?‘), and categorization (‘What was it?’, ‘Decode!’). CoCon consists of six games, and three of these games consisted of two or three sub-games. Thus, CoCon consists of a total of ten games. The tasks are ordered based on their difficulty levels. At first, users encountered a main character who introduced a whole story and explained how to play. Next, users entered cue-room where users choose game and checked their performances. Fig 2 depicted the schematic representation of the contents of CoCon. Fig 3 showed the main images of CoCon (the intro-screen, the main character, the cue-room and the screenshot of ‘stop signal’ game).

The most important feature of CoCon is its usage of specific algorithms. CoCon aimed to collect data about stable and practical functions that are demonstrated in common tasks such as learning. Therefore, it did not adopt simple and limited methods (e.g., one pass and one fail rule in every task). Instead, it adopted an adaptive staircase procedure. According to the adaptive staircase procedure, when users have successfully passed a specific number of trials (i.e., 1 or 3 successive passes), they proceed to the next level. However, if they fail a specific number of trials (i.e., 1 or 2 successive fails), they are redirected to the previous level. In particular, the final scores were computed as the ratio of correct to incorrect responses (range: -1–+1), and they were indicative of major cognitive control functions. A total of 4 composite index scores were extracted from the final scores: the SA, WM, CC, and CE indices are measures of sustained attention (composite score for “Find the circle!”), working memory (composite score for “Memory!”), cognitive control (composite score for “Stop! Signal!”), and cognitive (control) execution (composite scores for “What was it?” and “Decode!”), respectively. All indices were calculated to *T*-scores (*M* = 50, *SD* = 10) using means and SDs of the final scores of all the participants. In particular, means and SDs used to convert *T*-scores were computed for two separate developmental groups (birth years: 2007–2009 ‘younger group’ vs. 2003–2006 ‘older group’) to examine developmental trends. The means (SDs) of the younger group were -0.77 (2.2) for SA, -10.8 (11.23) for WM, 8.11 (5.37) for CC and 1.84 (2.52) for CE, and the means (SDs) of the older group were 0.15 (2.45) for SA, -3.05 (13.27) for WM, 9.61 (4.88) for CC and 2.4 (2.28) for CE.

All game-related data were imported into Microsoft EXCEL 2016 from the server. In this study, we collected data about not only game achievements index like SA, WM, CC, and CE but also ecological game behaviors (e.g., number of logins, reaction time for the first response, number of incomplete task exits, frequency with which game achievement levels were checked, and numbers of correct responses for practice tasks).

In development of the application, CoCon was developed using Unity 5.5.1f1 and C# (i.e., programming language). It took 7 months (December 1, 2017, to June 30, 2018) to develop CoCon. This includes the time that was taken for primary technical development, testing, and revision procedures. CoCon can be freely downloaded in the Google play store for the android smartphone user.

#### Korean Wechsler Intelligence Scale for Children-Fourth edition (K-WISC-IV-C)[32]

Ten core subtests of the K-WISC-IV-C were used to measure intelligence. The subtests (which are represented as scaled scores: *M* = 10, *SD* = 3) yield four composite indices (which are represented as standard scores: *M* = 100, *SD* = 15): Verbal Comprehension Index (VCI), Perceptual Reasoning Index (PRI), Working Memory Index (WMI), and Processing Speed Index (PSI). The Cronbach alpha of K-WISC-IV was .820

#### Korean version of the Stroop Color-Word Test for children [33]

The Stroop Color-Word Test assesses the ability to inhibit irrelevant information and select relevant responses [25]. We used the Korean version of the Stroop Color-Word Test for children in this study. Its reliability (Cronbach’s α) has been found to be 0.72 among the normative population and 0.73 among the clinical population [24]. The Korean norms for this test are available for those who are between the ages of 5 to 14 years. In this study, the correct number of color-words that were reported within a duration of 45 seconds for a duration of 45 seconds were used as measurement.

#### Korean version of the Color Trails Test (CTT) [34]

The original version of the CTT comprises two parts: the first part assesses the ability to sustain attention, and the second part assesses the ability to shift attention from one situation to another. The converted *T*-score of the time that was taken to complete both parts of the assessment were used as the composite score.

#### Executive Function Difficulty Questionnaire for Children and Adolescents [35]

The Executive Function Difficulty Questionnaire for Children and Adolescents [27] was used to assess perceived executive function. This questionnaire is composed of 40 items that assess four factors: planning/organizing difficulty, behavioral control difficulty, emotional control difficulty, and attention/concentration difficulty. Song [27] developed this questionnaire and validated it using exploratory and confirmatory factor analyses. Four factors were extracted, and the model fit indices for the four-factor model were acceptable (CFI = 0.892, TLI = 0.872, RMSEA = 0.049). The participants were required to indicate the severity of their perceived executive function difficulties (range of scores: 1–3). A higher score is indicative of greater executive function difficulties, and a lower score is indicative of strong executive functioning. The reliability coefficient (Cronbach’s α) of this scale was 0.897.

### Data collection

The participants were recruited through the internet or community advertisements. The examiners were students of a master’s program who were majoring in clinical child psychology. They had undergone a course entitled “Psychological Assessment” and completed rigorous practice administrations of the Korean WISC-IV Test, Stroop test, and CTT. An average duration of 90 minutes was required to complete all the individual neuropsychological tests. However, the total duration of gameplay that CoCon required varied substantially across participants. CoCon was developed based on a modified adaptive tracking algorithm to calculate reliable levels of performance. Therefore, participants with relatively better cognitive function took longer to complete the game than participants with poorer cognitive function. The participants were instructed to complete CoCon at home at a convenient time before they completed the neuropsychological tests. However, 17 out of the 100 participants completed CoCon on the same day on which they underwent the neuropsychological tests. They were instructed to individually complete CoCon within the testing environment before they were administered neuropsychological tests.

### Data analysis

Data analysis was conducted using version 21.1 of SPSS. Demographic data were analyzed by computing means, standard deviations, and frequencies. Bivariate correlation analysis was conducted to analyze the relationships between the scores yielded by Korean WISC-IV test, Stroop test, CTT, and Executive Function Difficulty Questionnaire, and game behavior.

A composite score for cognitive control was computed using scores that were yielded by the Working Memory Index (K-WISC-IV), the *T*-scores for the Stroop Color-Word Test, and reaction time for the CTT2 (the second part of the CTT). These three scores that were yielded by neuropsychological tests were converted into z-scores and summed to produce a composite score for cognitive control. The first (i.e., 25^th^ percentile) and third (i.e., 75^th^ percentile) percentiles were identified for the distribution of these scores. Participants who obtained scores that were below the 25^th^ percentile (cutoff score = -1.42) and above the 75^th^ percentile (cutoff score = 1.33) were classified as low and high cognitive control groups, respectively.

Independent-samples *t*-test was conducted to examine differences in game output and behavior between the high and low cognitive control groups. In addition, the receiver operating characteristics (ROC) curve and area under the curve (AUC) were inspected to examine whether game behaviors can distinguish between the two groups. Higher AUC values were considered to be indicative of a better ability of the model to distinguish between the high and low cognitive control groups. Typically, AUC values that are equal to or higher than 0.70 are indicative of a strong effect [36].

## Results

Table 1 presents descriptive statistics for demographic variables and performances on neuropsychological tests and game behavior (CoCon) data of all participants. The mean (SD) IQ score was computed for full scale IQ on the K-WISC-IV (Mean = 111.13, *SD* = 14.31). The mean IQ scores that were computed for performances on the subtests of the K-WISC-IV were indicative of average or above-average intelligence. Scores for performance on the Stroop test, CTT2, and Executive Function Difficulty Questionnaire were within the average range.

**Table 1:** Descriptive statistics for the study variables (*N* = 100).

Variables		*M* (*SD*)	Skewness	Kurtosis
	Age (years)	11.75 (1.40)	0.637	0.463
	School Grade	5.48 (1.52)	0.031	1.247
K-WISC-IV (IQ score)	Similarity	11.79 (3.09)	-0.173	-0.452
	Vocabulary	11.33 (3.07)	-0.009	-0.14
	Comprehension	11.42 (2.95)	-0.007	-0.008
	Block Design	11.91 (3.51)	0.110	-0.549
	Picture Concept	11.89 (2.97)	-0.237	-0.074
	Matrix Reasoning	11.78 (3.11)	-0.173	-0.294
	Digit Span	11.76 (3.43)	-0.189	-0.122
	Letter Number Sequencing	11.42 (3.01)	0.189	0.196
	Coding	9.53 (2.88)	0.226	0.082
	Symbol Search	10.55 (2.75)	-0.194	-0.086
	Verbal Comprehension	109.24 (15.53)	0.009	-0.131
	Perceptual Reasoning	113.00 (15.15)	-0.552	0.177
	Working Memory	109.00 (16.71)	-0.014	0.069
	Processing Speed	100.17 (14.11)	0.042	-0.297
	Full Scale (Intelligence Quotient)	111.13 (14.31)	-0.439	-0.103
Stroop test (T score)	Color-Word in 45 s ^a^	52.51 (10.77)	0.188	0.047
CTT (T score)	CTT1 (Reaction Time)	49.78 (9.75)	-0.907	0.764
	CTT2 (Reaction Time)	52.41 (8.32)	-1.123	1.647
EFDQ-C ^b^ (T score)	Planning/Organizing Difficulty	46.53 (8.76)	0.366	-0.396
	Behavior Control Difficulty	47.10 (8.80)	0.912	0.600
	Emotion Control Difficulty	47.73 (9.63)	0.999	0.720
	Attention/Concentration Difficulty	50.24 (9.35)	0.435	-0.742
CoCon Index Scores (T score)	Sustain Attention	50.00 (9.94)	-0.170	-0.812
	Working Memory	50.00 (9.94)	0.583	0.532
	Cognitive Control	50.00 (9.94)	-1.098	-0.765
	Cognitive Execution	49.99 (9.94)	-1.193	1.657
CoCon Ecological Behaviors (Frequency)	Number of “Diary” Visits ^c^	7.75 (5.69)	-0.506	0.478
	Number of Incomplete Task Exits	2.22 (2.77)	2.290	6.870
	Number of Logins	2.76 (2.09)	1.950	4.580
CoCon RT	RT to Complete Task (s)	1770.41 (590.03)	0.691	0.065
	RT for Correct Responses(s)	879.50 (286.97)	0.433	0.397
	RT for Incorrect Responses (s)	890.91 (379.76)	0.829	0.471

^a^ Number of correctly reported color-word pairs within 45 s.

^b^ Executive Function Difficulty Questionnaire for Children and Adolescents.

^c^ Frequency with which game achievement levels were checked.

Since the participants of this study were recruited from a community or through the internet, some of them had minor mental health problems. Specifically, a total of 8 out of 100 participants reported minor mental health problems (attention problems = 1, depressive mood = 1, and other undisclosed problems = 6), and they did not report any history of hospitalization or medication with regard to these problems. These 8 participants were comparable to the remaining 92 participants (i.e., those without any mental health problems) with regard to performance on the Stroop test and CTT, executive function, and game behaviors (i.e., Sustained Attention (SA), Working Memory (WM), Cognitive Control (CC), and Cognitive Execution (CE)). However, the 8 participants demonstrated poorer overall performance on the IQ test, *t*(98) = 2.313, *p* = 0.023, and obtained significantly lower scores on the WMI, *t*(98) = 2.747, *p* = 0.007, and PSI, *t*(98) = 2.418, *p* = 0.017, than the 92 participants who did not have mental health problems. The 4 participants with minor mental health problems were included in the low cognitive control group. The remaining 4 participants were not included in both the high cognitive control group and the low cognitive control group. In addition, another participant was suspected to be color blind, and was therefore not subjected to the Stroop test.

### Comparisons between performance on the CoCon and the standard cognitive assessments

#### Correlation analysis

CoCon index scores and several ecological game behaviors were significantly related to scores that were yielded by standard cognitive assessments (Table 2). SA (CoCon) was significantly related to subtest of the K-WISC-IV that require motor coordination and sustained attention abilities (Coding *r* = 0.328, *p <* .*001*, Processing speed index *r* = 0.337, *p <* .*001*). The WM (CoCon) was significantly related to the subtest of the K-WISC-IV that require short term memory or mental manipulation abilities (Digit span *r* = 0.458, *p <* .*001*, WMI *r* = 0.450, *p <* .*001*). The CC (CoCon) was significantly related to the subtests of the K-WISC-IV that require verbal processing abilities (Vocabulary *r* = 0.359, *p <* .*001*, VCI *r* = 0.304, *p* = 0.002) and the CE (CoCon) was significantly related to the subtests of K-WISC-IV that require executive function capabilities (Similarity *r* = 0.319, *p <* .*001*, Block design *r* = 0.369, *p <* .*001*). Further, the full scale IQ of the K-WISC-IV-C showed significant correlations with the WM (CoCon; *r* = 0.445, *p <* .*001*) and the CC (CoCon; *r* = 0.483, *p <* .*001*).

**Table 2:** Correlations between CoCon index scores and neuropsychological test scores (*N* = 100).

		1	2	3	4	5	6	7	8	9	10	11	12	13	14	15	16	17	18	19	20	21	22	23
1	IQ_Simi	1.000																						
2	IQ_Voca	.578^**^	1.000																					
3	IQ_Com	.523^**^	.622^**^	1.000																				
4	IQ_Block	.387^**^	0.163	0.051	1.000																			
5	IQ_SimPic	.247^*^	.234^*^	.235^*^	0.151	1.000																		
6	IQ_MatRea	0.046	0.079	(0.118)	.308^**^	0.184	1.000																	
7	IQ_DS	.356^**^	.470^**^	.277^**^	0.188	0.142	.348^**^	1.000																
8	IQ_LetterN	.337^**^	.398^**^	.269^**^	.215^*^	0.157	.316^**^	.612^**^	1.000															
9	IQ_Code	0.170	0.136	0.001	0.141	(0.051)	0.166	.199^*^	.254^*^	1.000														
10	IQ_Symbol	0.056	0.096	(0.017)	.319^**^	(0.050)	.248^*^	0.157	0.131	.459^**^	1.000													
11	IQ_VC	.831^**^	.868^**^	.839^**^	.235^*^	.276^**^	0.002	.437^**^	.393^**^	0.121	0.048	1.000												
12	IQ_PR	.339^**^	.228^*^	0.077	.743^**^	.610^**^	.712^**^	.326^**^	.329^**^	0.130	.258^**^	.249^*^	1.000											
13	IQ_WM	.387^**^	.483^**^	.306^**^	.225^*^	0.166	.372^**^	.911^**^	.882^**^	.242^*^	0.160	.463^**^	.366^**^	1.000										
14	IQ_PS	0.130	0.134	(0.008)	.260^**^	(0.062)	.237^*^	.208^*^	.223^*^	.862^**^	.846^**^	0.098	.219^*^	.234^*^	1.000									
15	IQ_Total	.675^**^	.681^**^	.513^**^	.546^**^	.402^**^	.474^**^	.696^**^	.672^**^	.424^**^	.413^**^	.733^**^	.690^**^	.761^**^	.486^**^	1.000								
16	StroopCW	.219^*^	.230^*^	(0.073)	.207^*^	0.013	0.172	.316^**^	.220^*^	.278^**^	.212^*^	0.153	0.191	.303^**^	.283^**^	.325^**^	1.000							
17	CTT1	.255^*^	.284^**^	0.148	0.190	0.081	.213^*^	0.084	.198^*^	0.190	.262^**^	.272^**^	.234^*^	0.157	.264^**^	.331^**^	0.185	1.000						
18	CTT2	.254^*^	.219^*^	0.084	.225^*^	0.013	0.134	0.050	.249^*^	.342^**^	.323^**^	.216^*^	0.189	0.159	.389^**^	.321^**^	.201^*^	.405^**^	1.000					
19	EFDQ-C^a^	(0.076)	(0.102)	(0.154)	(0.053)	(0.087)	-.202^*^	(0.143)	(0.186)	-.307^**^	(0.137)	(0.129)	(0.162)	(0.181)	-.264^**^	-.251^*^	(0.134)	-.203^*^	-.199^*^	1.000				
20	Cognitive Control^b^	.413^**^	.448^**^	0.153	.316^**^	0.092	.325^**^	.614^**^	.650^**^	.414^**^	.335^**^	.401^**^	.359^**^	.702^**^	.436^**^	.677^**^	.725^**^	.359^**^	.654^**^	-.247^*^	1.000			
21	Tscore_SA	.205^*^	.252^*^	0.130	.312^**^	0.120	0.171	0.184	0.157	.328^**^	.251^*^	.230^*^	.296^**^	0.188	.337^**^	.380^**^	.218^*^	0.032	0.194	-.310^**^	.288^**^	1.000		
22	Tscore_WM	.210^*^	.260^**^	0.035	.253^*^	0.026	.338^**^	.458^**^	.346^**^	.214^*^	.288^**^	.197^*^	.304^**^	.450^**^	.293^**^	.446^**^	.275^**^	0.055	.222^*^	(0.178)	.455^**^	.377^**^	1.000	
23	Tscore_CC	.257^**^	.359^**^	0.154	.309^**^	0.188	.234^*^	.352^**^	.280^**^	0.146	.357^**^	.304^**^	.350^**^	.357^**^	.293^**^	.483^**^	.296^**^	0.156	0.087	(0.125)	.356^**^	.367^**^	.453^**^	1.000
24	Tscore_CE	.319^**^	.225^*^	0.014	.369^**^	0.074	.339^**^	0.070	0.177	0.130	.287^**^	.224^*^	.383^**^	0.137	.239^*^	.361^**^	.299^**^	0.164	.250^*^	-.221^*^	.330^**^	.285^**^	.324^**^	.374^**^

*p < 0.05

**p < 0.01

***p < 0.001. () means ‘minus value’

^a^ Planning & organizing difficulty

^b^ Sum of z-scores for working memory index (K-WISC-IV-C), Stroop color-word test, and CTT2 scores.

**Fig 4:**
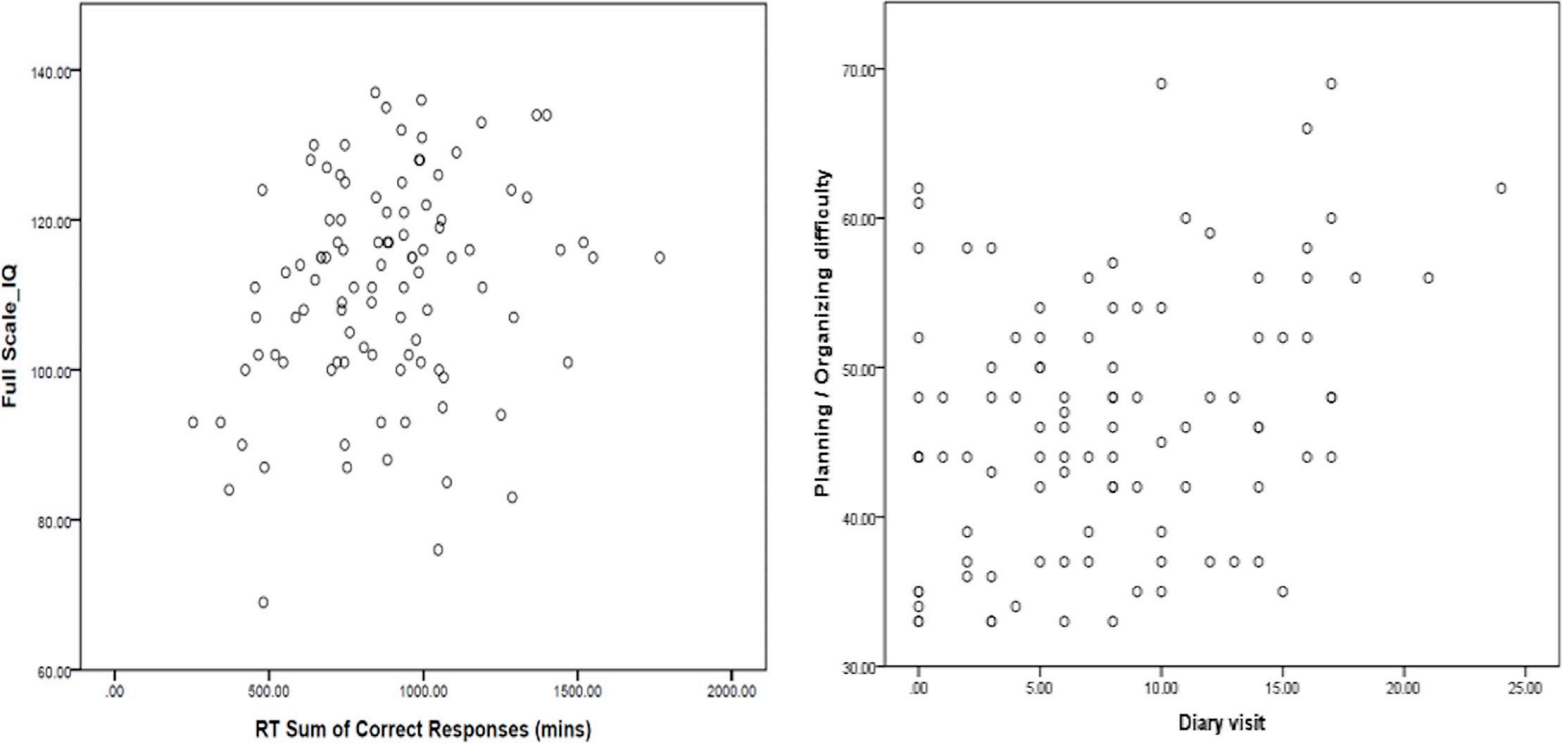
**A.** Scatterplots for the Relationships Between Full Scale IQ and RT sum of correct responses. **B.** Scatterplots for the Relationships Between Planning / Organizing difficulty and Diary Visit.

In addition, several game behaviors were significantly related to self-reported executive function difficulties. The frequency with which participants checked their game achievements (i.e., “Diary visit”) was significantly related to planning/organizing (*r* = 0.324, *p <* .*001*), behavior control (*r* = 0.215, *p* = 0.032), and attention/concentration difficulties (*r* = 0.291, *p* = 0.003). The number of incomplete task exits was significantly related to planning/organizing (*r* = 0.203, *p* = 0.043) and attention/concentration difficulties (*r* = 0.236, *p* = 0.018). The total reaction time for correct responses was significantly related to the full-scale IQ score (*r* = 0.250, *p* = 0.012) as well as performance on the WMI (*r* = 0.239, *p* = 0.016) and PSI (*r* = 0.203, *p* = 0.042). The scatterplots for the relationships between ecological variables and neuropsychological tests are presented in Fig 4A and Fig 4B.

To explore the unique contribution of neuropsychological test results in each game performance index, a linear regression analysis was conducted. Using the stepwise method, significant neuropsychological test variables to contribute each game performance index were extracted. Variables entered in each model were the four K-WISC-IV index-scores, the CTT I and II(reaction time T scores), the Stroop test(color word T score) and the self-reported executive function difficulty questionnaire(planning & organization difficulty T score). The results showed each game performance index had each different predictors of neuropsychological test variables (Table 3).

**Table 3:** Linear regression analysis investigating the association between game behavior indexes and standardized neuropsychological test variables.(stepwise method).

				Unstandardized Coefficients		Standardized Coefficients	*t*	*p-value*	Collinearity Statistics	
Factors		R square	Adjusted R square	B	Std. Error	Beta			Tolerance	VIF
SA(sustained attention) index	(Constant)	0.215	0.191	28.706	11.206		2.562	0.012		
	IQ_PS			0.170	0.067	0.241	2.520	0.013	0.899	1.112
	EF Plan			-0.245	0.107	-0.217	-2.285	0.025	0.920	1.087
	IQ_PR			0.140	0.061	0.213	2.278	0.025	0.941	1.062
WM(working memory) index	(Constant)	0.235	0.219	9.975	7.781		1.282	0.203		
	IQ_WM			0.238	0.055	0.399	4.355	0.000	0.948	1.055
	IQ_PS			0.139	0.065	0.198	2.158	0.033	0.948	1.055
CC(cognitive control) index	(Constant)	0.217	0.193	4.708	8.974		0.525	0.601		
	IQ_WM			0.139	0.059	0.233	2.354	0.021	0.844	1.185
	IQ_PR			0.147	0.065	0.224	2.268	0.026	0.848	1.179
	IQ_PS			0.135	0.067	0.191	2.026	0.046	0.927	1.079
CE (cognitive executive) index	(Constant)	0.2	0.184	13.418	7.549		1.777	0.079		
	IQ_PR			0.223	0.061	0.339	3.651	0.000	0.964	1.038
	Stroop CW			0.218	0.086	0.234	2.522	0.013	0.964	1.038

#### Group comparisons and classification

Table 4 shows the results of group comparisons (i.e., low vs. high cognitive control groups). The low cognitive control group included 26 participants (Boy = 12, Girl = 14) and the high cognitive control group included 25 participants (Boy = 17, Girl = 8). The high and low cognitive control groups did not differ significantly in gender ratios, χ^2^(1) = 2.48, *p* = 0.115, or age *t*(49) = 0.434, *p* = 0.666. The low and high cognitive control groups significantly differed on all four index scores (i.e., WM, CC, SA, and CE) that were yielded by the CoCon. However, the two groups did not differ in ecological game behaviors.

**Table 4:** Comparisons between the low and high cognitive control groups on CoCon index scores and neuropsychological test scores.

		Low Cognitive Control (N = 26)	High Cognitive Control (N = 25)	*t* (*p-value*)
Factors	Variables	*M* (*SD*)	*M* (*SD*)	
CoCon: Index Scores	SA score	45.98 (9.0)	51.85 (10.6)	**-2.14 (p = 0.038)**
	WM score	44.25 (9.9)	56.41 (10.5)	**-4.27 (p< .000)**
	CC score	46.22 (11.6)	54.29 (5.2)	**-3.22 (p = 0.003)**
	CE score	45.87 (12.6)	52.14 (5.0)	**-2.36 (p = 0.024)**
CoCon: Number of Ecological Behaviors	N of “Diary” visit	7.26 (6.51)	6.68 (4.98)	0.36 (p = 0.791)
	N of incomplete task exits	2.46 (2.23)	2.56 (3.55)	-0.12 (p = 0.906)
	Number of logins	2.57 (1.50)	2.08 (1.23)	1.26 (p = 0.211)
CoCon: Response time (RT)	RT to complete tasks (s)	1657.55(601.57)	1781.23(483.86)	-0.80 (p = 0.424)
	RT for Correct Responses(s)	789.63 (274.94)	935.36 (294.99)	-1.83(p = 0.074)
	RT for Incorrect Responses (s)	867.92 (434.23)	845.38 (245.29)	0.22 (p = 0.823)
K-WISC-IV	Similarity	10.19 (3.4)	13.40 (2.7)	**-3.72 (p = 0.001)**
	Vocabulary	9.23 (3.0)	13.00 (2.8)	**-4.60 (p< .000)**
	Comprehension	10.62 (3.4)	12.08 (2.4)	-1.77 (p = (0.084)
	Block Design	10.38 (3.9)	12.72 (2.9)	**-2.43 (p = 0.019**)
	Picture Concept	11.50 (2.8)	12.36 (2.8)	-1.10 (p = 0.276)
	Matrix Reasoning	10.38 (3.0)	12.84 (2.4)	**-3.20 (p = 0.002)**
	Digit Span	9.23 (3.5)	14.84 (2.4)	**-6.59 (p = 0.001)**
	Letter-Number Sequencing	9.00 (2.5)	14.20 (2.8)	**-7.11 (p< .000)**
	Coding	8.23 (2.3)	10.76 (3.4)	**-3.14 (p = 0.003)**
	Symbol Search	9.58 (2.0)	11.16 (2.5)	**-2.47 (p = 0.017)**
	VC index	100.19 (16.7)	117.08 (13.8)	**-3.96 (p < .000)**
	PR index	105.46 (15.4)	118.32 (10.6)	**-3.45 (p = 0.001)**
	WM index	94.88 (14.3)	126.00 (12.9)	**-8.14 (p< .000)**
	PS index	93.54 (10.4)	105.52 (15.8)	**-3.21 (p = 0.002)**
	Full Scale IQ	98.46 (13.9)	122.72 (9.8)	**-7.23 (p< .000)**
Stroop test	CWT score in 45 s	43.35 (9.1)	62.98 (8.5)	**-7.95 (p< .000)**
CTT	CTT1 (RT)	45.42 (11.4)	52.36 (8.6)	**-2.44 (p = 0.019)**
	CTT2 (RT)	44.73 (10.0)	58.00 (5.2)	**-5.98 (p< .000)**
EFDQ-C	Planning/Organizing Difficulty	49.27 (9.4)	42.80 (8.2)	**2.61 (p = 0.012)**

**Fig 5:**
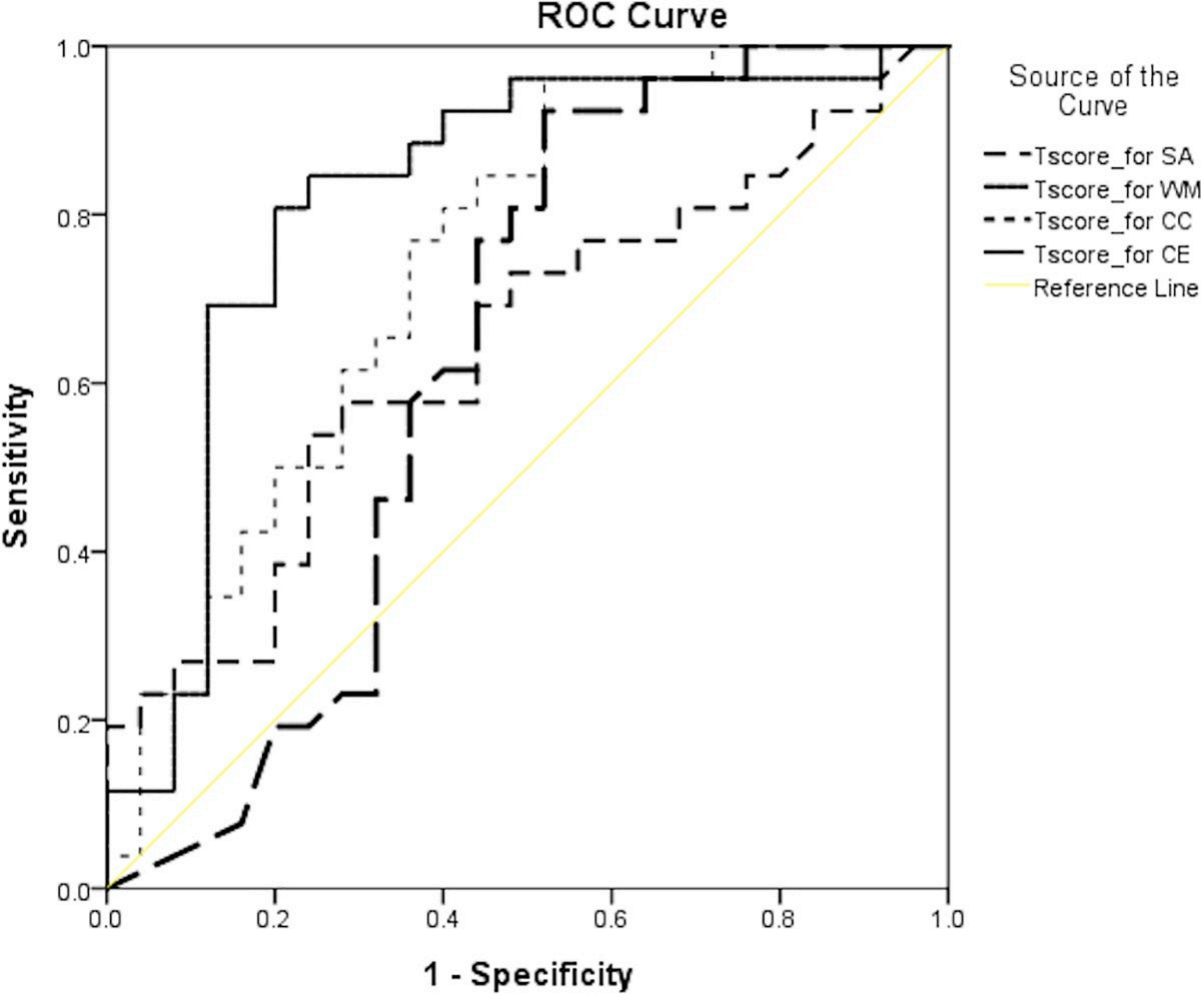
Receiver operating characteristics curves for the low and high cognitive control groups.

Based on the results of the ROC curve and AUC analyses, the scores of both groups were considered to be within the acceptable ranges. The AUC value for WM, CC, SA, and CE (CoCon) were 0.821, 0.715, 0.667, and 0.648, respectively (Fig 5).

## Discussion

This study aimed to examine whether a mobile game application, namely, CoCon, which had been developed using algorithms that were based on the modified adaptive staircase method, could serve as a reliable and valid screening measure of cognitive control among children and adolescents. Additionally, the study examined whether CoCon had any additional benefits when compared to conventional psychological assessments. To address this objective, we analyzed the relationship between various game behaviors that the CoCon entailed and scores that were yielded by standardized neuropsychological tests.

The results of correlation analysis revealed that all the four index scores that were yielded by CoCon were mild to moderately correlated with subtests, index scores, and full-scale IQ scores as well as performance on the CTT test, the Stroop test and self-reported executive difficulty. These results suggest that CoCon was able to efficiently screen cognitive control among children and adolescents. Impressively, CoCon index scores correlated against criterion measures in a manner that was consistent with the theoretical framework of each index scores. For example, the WM (CoCon) index score was significantly correlated with performance on the digit span test and WMI score of the K-WISC-IV. CE (CoCon) was significantly related to the subtests of the K-WISC-IV-C that required higher cognitive functions such as executive function (e.g., block design, matrix reasoning) and PSI. In particular, the CC index score (CoCon) emerged as a comprehensive index score that was related with more variables. These results supported the primary hypothesis of this study that CoCon will serve as a reliable and valid screening measure of cognitive control, which is a fundamental function that is necessary for engagement in adaptive human behaviors. Unexpectedly, the CC index score was significantly related to the VCI of K-WISC-IV. These results might be attributable to the fact that the tasks included in the CC index required the auditory processing abilities such as a sensitivity to the auditory stimuli or auditory discrimination ability. Usually, the sensitivity to the auditory stimuli and the auditory discrimination ability are regarded as to correlate with performance on the vocabulary test of the K-WISC-IV. In addition, the SA and the CE index scores that were yielded by CoCon were significantly related to self-reported executive function difficulties. The SA and CE index scores were significantly related to self-reported difficulties in planning/organizing. In addition, the linear multiple regression analysis showed similar and more clear findings to support that CoCon indexes could work as a valid and reliable screening tool of cognitive control function. In particular, these findings were worth in the fact that CoCon was able to assess cognitive control as validly as standard neuropsychological tests even though the users completed the tasks as per their own convenience and with wide variability in time and place of completion and login restrictions.

Second, the patterns of relationships that emerged between ecological game behaviors and neuropsychological test scores were different from those that emerged between CoCon index scores and the criterion variables. Ecological game behaviors such as the frequency with which game achievements were checked (i.e., diary visits) and the number of incomplete task exits were significantly related to self-reported executive function difficulties. However, ecological game behaviors were not significantly related to scores that were yielded by performance-based neuropsychological tests. These findings suggest that ecological game behaviors are related to subjective rather than objective experiences of cognitive control, the latter of which is assessed by standardized neuropsychological tests. Greater levels of perceived cognitive function difficulties were associated with a greater frequency of checking one’s performance and incomplete task exits.

According to these findings, some children and adolescents with subjectively perceived difficulties in cognitive function may demonstrate less efficient behaviors that lead to undesirable outcomes even though they possess good cognitive abilities. Toplak, West, and Stanovich [37] have differentiated between subjectively experienced and objectively assessed cognitive function by referring to the discrepancy between performance-based and self-reported measures of executive function. Toplak et al. [37] have noted that performance-based and self-report measures of executive function assess different mental constructs. Therefore, they have suggested that, in clinical contexts, it is necessary to use a comprehensive assessment that assesses both these constructs in order to obtain valuable information to provide differential intervention based on test results. If traditional testing environments, which entail supervision and structure, are not necessary prerequisites for the rendition of reliable and valid data, then mobile game application can be used to assess multi-dimensional constructs and efficiently integrate various characteristics.

Finally, group comparisons on the composite score for cognitive control revealed that the high cognitive control group demonstrated better performance on each index that is measured by CoCon than the low cognitive control group. In particular, although the low cognitive control groups were recruited from the community and self-selected group as well as not clinically diagnosed group, it was impressive to present significant group differences in the game performance indexes. The WM and CC index scores emerged as the more powerful index scores that could discriminate between varied levels of cognitive control. The tests that assess WM and CC involve visual and auditory modalities or multimodalities. On the other hand, the tests that assess SA and CE involve only visual modalities. This result suggests that a multidimensional assessment can be used to quickly and efficiently measure a set of attributes as long as the multidimensionality of the assessment does not adversely affect the validity of the assessment.

In sum, the present findings revealed that a mobile game application based assessment is a reliable and valid screening measure of the cognitive control in children and adolescents. Specifically, even though the participants completed the mobile game ‘CoCon’ in their natural habitats, the resultant scores were comparable to those that were yielded by standard neuropsychological tests.

It was very impressive that, even though the CoCon is a game that anyone can download from play stores and play as many games as they want in any place and at an anytime, the index scores that were yielded by the CoCon were significantly related to various cognitive control functions and differentiated between the high and low cognitive control groups. Ultimately, the present findings suggest the possibility that mobile games that use advanced technology and sophisticated psychological strategies can serve as a new and expanded platform for the administration of psychological assessments.

This study has several limitations. First, the sample had some limitations to represent the general children and adolescent group. The sample included 8 participants who had minor psychological problems. In addition, this sample’s IQ scores were relatively high (mean full-scale IQ was 111). Nevertheless, our sample was adequately representative of the demographic group of children and adolescents. In other words, their means and standard deviations for neuropsychological variables corresponded to normal levels and the scores were normally distributed. However, a sample size of 100 is not adequate enough to permit the generalizations of the findings to a larger community sample. Therefore, future studies must test the present findings using larger samples.

Second, this study did not include a group of individuals with diagnosable behavior problems such as ADHD, conduct disorder, or Autism Spectrum Disorder which are characterized by serious cognitive control deficits. Consequently, we didn’t get any significant group differences in the ecological game behaviors like the login frequency and uncompleted exit frequency. The primary objective of this study was to verify CoCon as a screening tool for cognitive control through comparison with conventional standardized neuropsychological assessments among the general community samples as a starting study. However, in the following study, it is necessary to include data from the groups with serious behavior problems to extend the usage of CoCon to clinical populations with serious cognitive control problems.
